# A Pipeline to Assess Disease-Associated Haplotypes in Repeat Expansion Disorders: The Example of MJD/SCA3 *Locus*

**DOI:** 10.3389/fgene.2019.00038

**Published:** 2019-02-05

**Authors:** Inês P. D. Costa, Beatriz C. Almeida, Jorge Sequeiros, António Amorim, Sandra Martins

**Affiliations:** ^1^i3S – Instituto de Investigação e Inovação em Saúde, Universidade do Porto, Porto, Portugal; ^2^IPATIMUP – Institute of Molecular Pathology and Immunology of the University of Porto, Porto, Portugal; ^3^Faculdade de Ciências da Universidade do Porto, Porto, Portugal; ^4^IBMC – Instituto de Biologia Molecular e Celular, Universidade do Porto, Porto, Portugal; ^5^ICBAS – Instituto de Ciências Biomédicas Abel Salazar, Universidade do Porto, Porto, Portugal

**Keywords:** haplotype, repeat instability, CAG expansion, mutation origin, Machado-Joseph disease, SCA3, SNP, STR

## Abstract

At least 40 human diseases are associated with repeat expansions; yet, the mutational origin and instability mechanisms remain unknown for most of them. Previously, genetic epidemiology and predisposing backgrounds for the instability of some expanding *loci* have been studied in different populations through the analysis of diversity flanking the respective pathogenic repeats. Here, we aimed at developing a pipeline to assess disease-associated haplotypes at oligonucleotide repeat *loci*, combining analysis of single nucleotide polymorphisms (SNPs) and short tandem repeats (STRs). Machado-Joseph disease (MJD/SCA3), the most frequent dominant ataxia worldwide, was used as an example of a detailed procedure. Thus, to identify genetic backgrounds that segregate with expanded/mutated alleles in MJD, we selected a set of 26 SNPs and 7 STRs flanking the causative CAG repeat. Key criteria and steps for this selection are described, and included (1) haplotype blocks minimizing the occurrence of recombination (for SNPs); and (2) match scores to increase potential for polymorphic information content of repetitive sequences found in Tandem Repeats Finder (for STRs). To directly assess SNP haplotypes in phase with MJD expansions, we optimized a strategy with preferential amplification of normal over expanded alleles, in addition to SNP allele-specific amplifications; this allowed the identification of disease-associated SNP haplotypes, even when only the proband is available in a given family. To infer STR haplotypes, we optimized a multiplex PCR, including 7 STRs plus the MJD_CAG repeat, followed by analysis of segregation or the use of the PHASE software. This protocol is a ready-to-use tool to assess MJD haplotypes in different populations. The pipeline designed can be used to assess disease-associated haplotypes in other repeat-expansion diseases. This should be of great utility to study (1) genetic epidemiology (population-of-origin, age and spreading routes of mutations) and (2) mechanisms responsible for *de novo* expansions, in these neurological diseases; (3) to detect predisposing haplotypes and (4) phenotype modifiers; (5) to help solving cases of apparent homoallelism (two same-size normal alleles) in diagnosis; and (6) to identify the best targets for the development of allele-specific therapies in ethnically diverse patient populations.

## Introduction

Repetitive DNA sequences with a capacity to expand (sometimes up to hundreds or thousands of repeats) are found in the human genome in non-coding and exonic regions. They are currently known to be associated with approximately 40 human diseases (reviewed in Paulson, 2018). Trinucleotide repeats were the first to be identified, but an intensive search over the last decades has shown tetra-, penta- and hexanucleotide repeats also expanding above a normal polymorphic range in some human subjects (Paulson, 2018). Most of the repeat-associated disorders manifest with neurological, neuropsychiatric or neuromuscular symptoms. Among them are the spinocerebellar ataxias (SCA1, DRPLA, SCA2, MJD/SCA3, SCA6, SCA7, SCA8, SCA10, SCA12, SCA17, SCA31, and SCA36), Huntington’s disease, myotonic dystrophy, spinal-bulbar muscular atrophy and fragile X syndrome. While each of these diseases is rare worldwide, together they are one of the commonest causes of hereditary neurological pathology (Sequeiros et al., 2011; Paulson, 2018).

Non-human primates have short repeat alleles at all these *loci* (Andrés et al., 2004), which implies that expansion into pathogenic ranges occurred after Homo-Pan split. If we are able to identify the genetic backgrounds where *de novo* expansions occurred (place-of-birth for these mutations), many new possibilities will open for the study of mutational origins and spread (Sequeiros et al., 2011; Obayashi et al., 2015; Lee et al., 2016; Bampi et al., 2017; Kay et al., 2017), as well as mechanisms of repeat instability (Martins et al., 2006; Falush, 2009; Warby et al., 2009; Ramos et al., 2010) and genetic modifiers for these diseases (Filippova et al., 2001; Libby et al., 2008; Becanovic et al., 2015).

To characterize the genetic background of expanded alleles, polymorphic markers are crucial given their capacity to distinguish alleles identical-by-state. Single nucleotide polymorphisms (SNPs) are the most common source of genetic variation in the human genome, but their biallelic nature reduces their informativeness. Short tandem repeats (STRs) have a very high polymorphism information content (PIC), but their mutation rate ranging 10^-6^–10^-2^ *per* locus *per* generation (Fan and Chu, 2007) causes a high rate of recurrence. A combined stepwise analysis with both SNPs and STRs may then be the key to overcome both problems.

In Machado-Joseph disease (MJD/SCA3), SNPs and STRs have been used mainly to study genetic epidemiology in this dominant ataxia, the most frequent SCA worldwide (Martins and Sequeiros, 2018); however, there is also evidence on the importance of extending haplotype analyses to study MJD instability (Martins et al., 2008). MJD is caused by an expanded (CAG)_n_ in exon 10 of the *ATXN3* gene (14q32.12) (Kawaguchi et al., 1994). As in other SCAs, repeat instability of mutated/expanded alleles has received enormous attention, due to its importance in the clinical phenomenon of anticipation: the earlier age-at-onset (AO) and more severe symptoms in successive generations, as the repeat number tends to increase upon transmission to offspring, and repeat size correlates inversely with AO. Despite its importance, instability is not yet fully understood in MJD or other repeat-associated disorders; only a few modifiers have been identified (McGinty and Mirkin, 2018). In MJD, in addition to the length of the initial repeat tract and the gender and age of the transmitting parent (Maciel et al., 1995; Maruyama et al., 1995; Souza et al., 2016), SNP rs12895357 near the CAG repeat has been shown to affect repeat instability, the genotype (CAG)_exp_-C/(CAG)_normal_-G of the transmitting parent being associated with increased instability (Igarashi et al., 1996; Maciel et al., 1999; Martins et al., 2008).

Given the importance of haplotype analyses (including SNPs and STRs) to perform a comprehensive study of oligonucleotide repeat-related diseases, we designed a strategy to identify disease-associated haplotypes and show here the example of this approach to analyse the *ATXN3* locus.

## Materials and Methods

### Samples

We optimized a protocol with DNA samples extracted from saliva, buccal swab and peripheral blood through different techniques, by using the QIAamp^®^ DNA Blood Mini kit, the Citogen^®^Blood kit, the Chelex100 chelating resin, as well as through the standard method of salting-out. We also tested some DNA samples stored for more than two decades at 4°C. This study was carried out with anonymized DNA samples available in our laboratory, in accordance with the recommendations of international guidelines. Previous written informed consent was obtained from all subjects to use their DNA samples for research purposes, in accordance with the Declaration of Helsinki. DNA quantification was performed with Nanodrop, to prepare aliquots with a final DNA concentration of 7.5 ng/μL.

### Selection of Polymorphic Markers

We selected a set of 26 SNPs within a region of 4 kb encompassing the (CAG)_n_, based on (1) minor allele frequencies (MAF > 5%; Ensembl ^1^); (2) recombination hotspots (within the same haplotype block; The International Genome Sample Resource and The 1000 Genomes Project); and (3) sequence alignments of previously analyzed patients (to include SNPs that discriminate MJD lineages (Martins et al., 2012; Ogun et al., 2015); Supplementary Table S1).

To select STRs, we used the Tandem Repeats Finder tool^2^ and the following criteria for putative polymorphic repeats: consensus size of 2–5 bp; copy number above 7; matches above 80%; and proximity to the (CAG)_n_. This search was done within the human reference *ATXN3* and 250 kb up and downstream to it (NG_008198.2). Seven STRs were selected, based on their score (>8.5): one tetra, two tri and four dinucleotide repeats, less than 223 kb from the (CAG)_n_, thus reducing the likelihood of recombination (Table 1). Three of these STRs (TAT223, AC21 and GT190) have been analyzed in previous haplotype studies in several MJD populations, which will be useful for future comparisons (Martins et al., 2007).

**TABLE 1:** STRs selected for MJD haplotype analysis, respective distances from the (CAG)_n_ and primers used for genotyping. F - Forward primer; R - Reverse primer.

Primers	Distance from the (CAG)_n_	Primer sequence (5′–3′)
TAT223_F^∗1^	223 kb	CCACACTTCCTTTGGACCAT
TAT223_R		GGTAGGCACCAGCTACTTGGG
GT199_F	199 kb	TTACTGGGTAGGATATACATTCC
GT199_R^∗1^		CAGCCTTCCCCCGAGTCC
ATA194_F	194 kb	CCTTATCTAACCTCCTACATCTCAGC
ATA194_R^∗2^		GCAGGGCAGGCAATGAAACACG
MJD52_F		CCAGTGACTACTTTGATTCG
CAG_R^∗3^		GTGTGAAGGTAGCGAACATGATG
AC21_F^∗1^	21 kb	CTTCAGCTCAAATGCTATCAAAC
AC21_R		CAAGGATGGCTAGTGCAGAAAT
AAAC123_F	123 kb	CAGATGGGATAGGCCACAGT
AAAC123_R^∗1^		AGTGGAGGCTTCAACCTGTT
GT190_F^∗4^	190 kb	GAGGGGACCTGGCCTACTAC
GT190_R		ACCTACAGTAACACACTTTGCAC
AC190_F^∗2^	191 kb	CTGGGAGGAGGAGGGTACAA
AC190_R		AACCCTGACTCAACTCTCGG

*Fluorescent labels ^∗1^FAM (6-carboxufluorescein); ^∗2^NED; ^∗3^PET; and ^∗4^VIC*

### Primer Design

We used the online software Primer3Plus to design specific primers, to amplify and sequence previous selected polymorphic markers, in addition to the MJD_CAG repetitive region (Table 2). Next, the alignment tool BLAST was used to guarantee the specificity of the designed primers: at maximum three mismatches (or two at 3′ end) were allowed if homologous sequences were observed. Finally, the occurrence of hairpins and primer-dimers (including self-dimers) was tested with AutoDimer (Vallone and Butler, 2004).

**TABLE 2:** Primers designed to amplify and sequence a 4 kb flanking region of the *ATXN3*_CAG repeat. F - Forward primer; R - Reverse primer.

Name	Primer sequence (5′–3′)
MJDcloF_F	CAATTATTGGCCTTTCTGAACC
MJD52_F	CCAGTGACTACTTTGATTCG
MJD653_R	GCAAATGAGTGTTGGTTTATAGACCC
MJD716_F	ACAGAGTCTCGCTCTGTCGCCCAG
MJD1260_R	GCTGTCTGAAACATTCAAAAGTGAAG
MJD7a_R	TGCTCCTTAATCCAGGGAAATTTAG
MJD1342_F	CCACCAGTTCAGGAGCACTT
MJD1396_F	TCATGTTCGCTACCTTCACACT
MJD2109_F	GAGTTACTTTCCAGGTCTCGG
MJD2129_R	CCGAGACCTGGAAAGTAACTC
MJD2552_F	GATCCAGCAGTCCCAATCATGTA
MJD2646_R	TGCCTGGTCAGCTATAAGCA
MJD2942_F	TGGACACGGTGGCTTACGCCT
MJD3417_F	CTGGGCTGGGTGGCGGTGGCTCA
MJD3936C_R	CTAAAGGTTTTTATCTTGCTAGAC
MJDcloR_R	AGCCTTCTCTAACACCACCTTGG

### SNP Haplotypes

Genotyping of the SNPs was done with amplification of three fragments, not including the CAG tract, which allowed equal amplification of normal and expanded alleles. Reactions were performed using the following primers, annealing conditions, extension time, and number of cycles: MJDcloF-MJD1260R (62°C for 90 s; 90 s; 33 cycles); MJD1342F-MJD2646R (59°C for 90 s; 120 s; 35 cycles); MJD2552F-MJDcloR (61°C for 90 s; 120 s; 35 cycles). Amplification reactions were done in a total volume of 10 μL, with 0.2 μM of each primer, 1x of Taq PCR Master Mix Kit Qiagen^®^, 0.5x of Q-Solution for Qiagen^®^, and 15 ng DNA.

Phase of SNPs on expanded chromosomes was assessed through (1) amplification of two fragments encompassing the CAG repeat and either up or downstream regions (which resulted in the overrepresentation of normal alleles) and (2) allele-specific amplification of rs7142326 (amplicon length of 1384 bp). Reactions were performed using the following primers, annealing conditions, extension time and cycles: MJDcloF_F-MJD7a_R (60°C for 90 s; 120 s; 35 cycles), MJD52_F-MJD2646_R (58°C for 90 s; 120 s; 20 cycles; plus, 57°C for 90 s; 120 s; 20 cycles) and MJD1342_F-MJD3936C_R (57°C for 90 s; 120 s; 40 cycles). PCRs included 0.2 μM of each primer, 1x of Taq PCR Master Mix Kit Qiagen^®^, 0.5x of Q-Solution for Qiagen^®^and 15 ng DNA, in a final volume of 10 μL.

To sequence the amplified fragments, we started by purification with thermosensitive Alkaline phosphatase: Exonuclease I, ExoFastAP (Thermo Scientific) (1:5) at 37°C for 15 min, followed by 15 min at 80°C to inactivate the enzyme. Sequence reactions were done with 0.5 μL BigDye^®^Terminator Cycle kit (Applied Biosystems), according to manufacturer’s instructions.

A final purification of DNA was performed using a cross-linked dextran matrix (Illustra™ Sephadex™ G-50, GE Healthcare), with centrifugation for 4 min at 4400 rpm. After loading the sequencing product, the same conditions of centrifugation resulted in a deposit containing the final product, ran in an ABI PRISM 3130x/Genetic Analyzer (Applied Biosystems) with Hi-Di™ formamide.

### STR Haplotypes

STRs were all amplified together with the CAG repeat, in a multiplex PCR reaction, in a final volume of 10 μL, using 0.25 μM (AAAC123, AC21, GT199, and GT190), 0.125 μM (TAT223, ATA194, and AC190), and 0.2 μM (CAG, and MJD52_F) of each primer, 1x of Taq PCR Master Mix Kit Qiagen^®^, 0.5x of Q-Solution for Qiagen^®^, and 7.5 ng DNA. The initial denaturation was performed at 95°C for 15 min, followed by 35 cycles of denaturation at 94°C for 30 s, annealing at 62°C for 90 s and extension at 72°C for 60 s; with a final extension at 70°C for 30 min. Analysis of fragment length was performed with ABI PRISM 3130x/Genetic Analyzer (Applied Biosystems). A mix of GeneScan™ 500 LIZ™ size standard (Thermo Scientific):Hi-Di™ formamide (Applied Biosystems) (1:20) was added to 2 μL of PCR product and run in matrix G5, analyzed with GeneMapper v4.0. Allelic phases associated with the expansion were assessed by segregation and bioinformatically, using PHASE v.2.1.1 whenever DNA samples from relatives were not available.

## Protocol Design and Applications

Analysis of the genetic background of expanded alleles at repetitive *loci* is important for a comprehensive study of repeat-associated neurological disorders. Most approaches, however, are rather *ad hoc* and lack a strategy to get the most from such valuable SNP and/or STR data from patients. Thus, we designed a pipeline to characterize the haplotype background of repetitive disease-associated *loci* that can be used to study any of these neurological disorders; a step-by-step protocol, optimized to assess genetic backgrounds of MJD patients, is here detailed as an example.

### Process Overview

**FIGURE 1:**
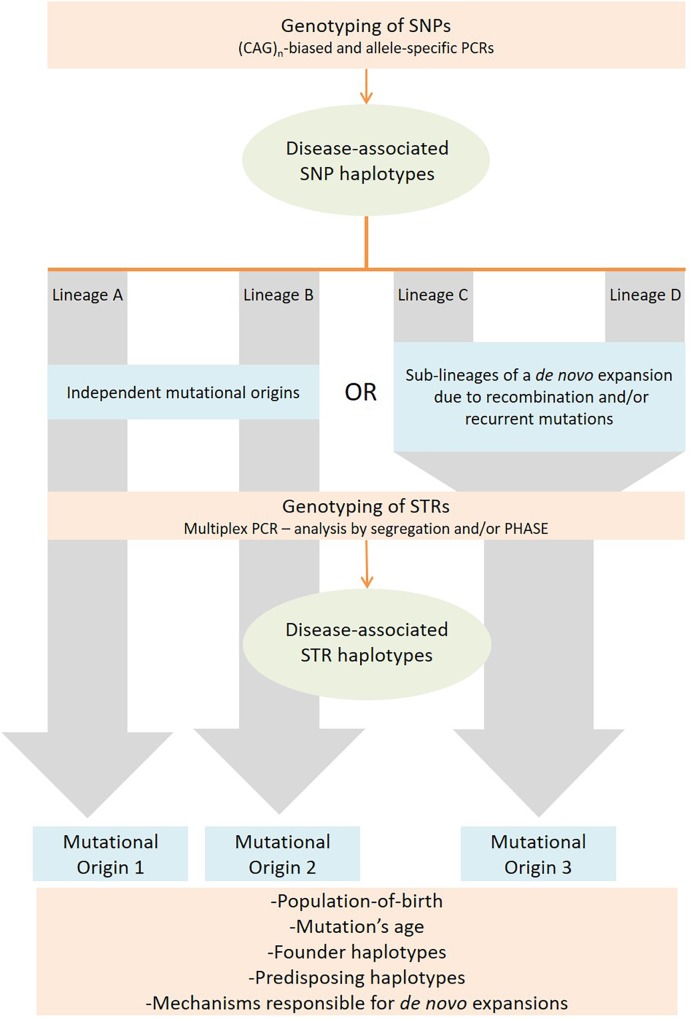
Strategy followed to identify mutational origins in repeat-associated diseases by assessing SNP and STR haplotype backgrounds associated to expanded alleles.

Our strategy focused first on the selection of SNPs to identify stable genetic backgrounds, defining lineages. Given the low mutation rate of SNPs (∼2.5 × 10^-8^), events on the origin of these polymorphic markers are considered unique during the evolution of a species (Nachman and Crowell, 2000). It is important that SNPs are selected within a distance to the pathogenic repeat small enough to lie within a single haplotype block, thus avoiding recombination to play a relevant role on the lineages identified. Next, analysis of STRs allows differentiating a high number of haplotypes inside each lineage. Fast-evolving STRs flanking the disease *locus* should have a high potential to be pure, simple stretches, in order to be reliable as molecular clocks. By following this two-level strategy (STRs analyzed within stable SNP lineages), it is possible to achieve a high discrimination power, not compromising the discrimination of haplotypes identical-by-state (not by-descent) (Figure 1).

### Protocol to Identify MJD Haplotype Backgrounds

**FIGURE 2:**
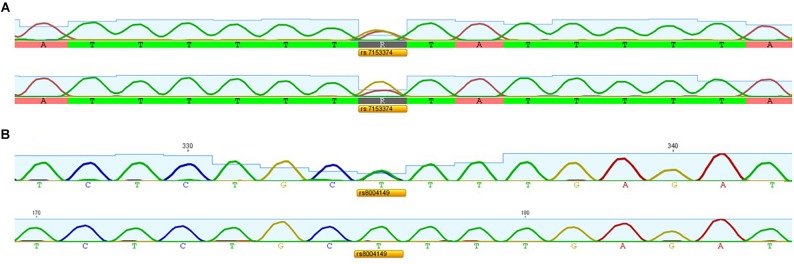
Electropherograms showing **(A)** SNP rs7153374 genotyped by a standard PCR *versus* (CAG)_n_-biased PCR and **(B)** SNP rs8004149 genotyped by a standard PCR *versus* allele-specific PCR.

**FIGURE 3:**
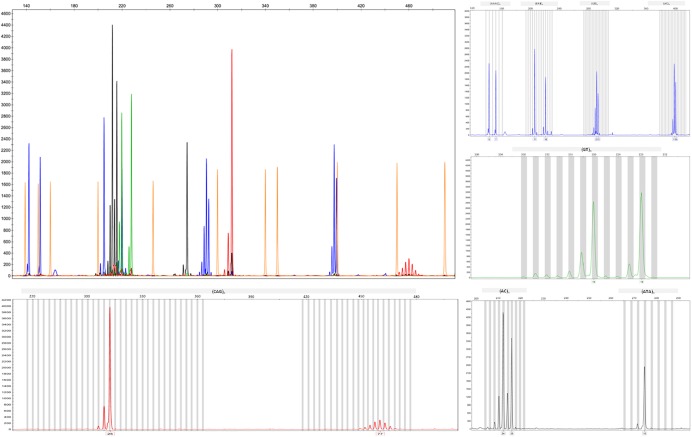
Electropherograms showing amplified products from the optimized multiplex PCR for the analysis of the CAG repeat of *ATXN3* and seven flanking STRs.

We applied the strategy described to optimize a protocol to assess disease-associated haplotypes in MJD. While sequence, frequency and population data are widely available for SNPs in databases such as Ensembl and The 1000 Genomes Project, the search for STRs must rely on the potential PIC that a repetitive sequence harbors, since there are not many data available regarding allele frequencies and repeat configuration of non-deleterious STRs. For this reason, we selected potential STRs from Tandem Repeat Finder and tested their heterozygosity value by genotyping a set of random samples. After confirming their high potential to discriminate alleles identical-by-state, we sequenced at least one allele size *per* STR from two major ethnic groups: Europeans and Asians. Further analyses included exclusively pure STRs (or STRs with regular repeat configurations), without any additional source of size variation (such as indels or other tandem repeats) within the amplicon that includes the STR of interest. While optimizing the protocol, we performed three standard PCRs, to obtain SNP genotypes of MJD patients; followed by two PCRs encompassing the (CAG)_n_ (Figure 2A) and an allele-specific PCR (Figure 2B), to assess alleles that segregated with the MJD expansion. This way, even in families with a single DNA sample available from the proband, we were able to infer directly alleles in *cis* with the expansion (i.e., lineages). For genotyping of STRs, we optimized a single multiplex reaction, to amplify all 7 STRs and the MJD_CAG repeat together (Figure 3), this way reducing quantity of DNA, time, reagents and sample’s manipulation.

We validated our pipeline for MJD haplotyping by testing the optimized protocol in a randomly selected subset of 100 MJD families from our large cohort. Genotypes of SNPs and STRs were obtained in 92 families, a successful rate for SNP and STR amplification of 92% - taking into account the long-term storage of most DNA samples analyzed (most for over two decades), we considered 8% a low failure rate. We have also estimated success rate for haplotype inference, given the importance of assessing allelic phase of polymorphisms segregating with the pathogenic expansion: following our strategy of SNP allele-specific amplification, MJD lineages were accurately identified in all 92 families; as for flanking STRs, we were able to reconstruct haplotypes in 85% of the families genotyped (78/92); of note is the fact that 48% (44/92) of the families were composed solely by the proband and that in other 15% (14/92) only a single relative was available. Therefore, we may conclude that, following this protocol, complete extended haplotypes (SNPs-STRs) can be inferred in a larger number of MJD families, even in very small families or isolated patients.

### Usefulness and Perspectives of Application

1. **Genetic epidemiology.** Geographical differences in disease prevalence may be explained by haplotype studies, as shown for Huntington disease (HD), with the highest risk HD haplogroups being found in Europe, while absent in East Asia (Warby et al., 2011). In MJD two *de novo* expansions seem to have occurred, and, so far, its presence in remote and ethnically diverse populations has been explained by genetic drift and founder effects (Gaspar et al., 2001; Martins et al., 2007, 2012; Ogun et al., 2015); This optimized protocol could clarify remaining questions about the origins and history of MJD mutations. By following the same pipeline to study other expanding *loci*, one could understand better disease prevalence and provide a clearer scenario about the occurrence of *de novo* expansions (Venkatesh et al., 2018).
2. **Mechanisms of *de novo* expansion.** Analysis of SNPs and STRs flanking *ATXN3* suggested a multistep mutation mechanism for the evolution of the CAG repeat responsible for MJD (Martins et al., 2006). Also based on the analysis of flanking haplotypes, other authors explained the origin of a rare intermediate MJD allele (45 CAGs) after a gene conversion event (Mittal et al., 2005). In other expansion diseases, risk SNP haplotypes seem to predispose to large jumps, namely large expansions into the pathogenic range in *HTT* (responsible for HD) (Warby et al., 2009) and in *C9orf72* (the most common known genetic cause of amyotrophic lateral sclerosis and frontotemporal lobar degeneration) (Xi et al., 2015) or large contractions into the normal range in fragile X (Maia et al., 2017).
3. **Instability of expanded alleles.** Once expanded, the haplotype background seems to affect (CAG)_n_ intergenerational instability, since *cis*-elements in different haplotypes may regulate instability at that *locus.* In paternal transmission of MJD, opposite biases towards further expansion or contraction have been observed on specific SNP backgrounds: TTACAC *versus* GTGGCA haplotypes (Martins et al., 2008); this shows the relevance of further exploring the effect of flanking sequences on repeat instability. Haplotypes can serve as tags of *cis* or *trans* elements influencing *de novo* expansion or biasing towards further expansion or contraction, but can also have a direct effect. In the SCA7 gene, CTCF binding-sites *cis* to the expansion were shown to regulate *ATXN7*_CAG hyperinstability (Libby et al., 2003, 2008). Also, a haplotype study in fragile X has shown the direct influence of a flanking SNP on the replication of CGG repeats, due to its location within a medium reiterative element 1B sequence, proved to affect chromatin structure (Ennis et al., 2007). Through the identification of haplotypes, either with direct or indirect effect on repeat instability, we can improve genetic counseling, by predicting further expansion or contraction in offspring, but also understand better the mechanisms behind such events.
4. **Phenotype modifiers.** Expanded repeat disorders frequently show an inverse correlation between expansion size and AO; however, this correlation explains only part of AO variation (Du Montcel et al., 2014). Haplotype background may play a direct role in disease expressivity; even after correcting for the known effect of expansion size; this is the case when a given SNP lies in the promoter or regulatory sequences of repeat-associated *loci*, which can affect binding of transcription factors, thus altering gene expression and disease presentation. In HD, SNP rs13102260 was identified as a modifier of AO. This SNP is located in a NF-κB binding site that regulates *HTT* promoter transcriptional activity. *In vitro* data showed a direct effect of rs13102260 on NF-κB binding and huntingtin expression, this way affecting disease presentation (Becanovic et al., 2015).
5. **Clinical laboratory diagnosis.** The analysis of polymorphisms flanking the disease-causing repeat may also be helpful in diagnosis. A new genetic tool using 13 STRs flanking the expansion repeat has been proposed to help fragile X detection, avoiding ambiguity due to allele dropout (Rajan-Babu et al., 2017). Analysis of genetic markers is also important for predictive testing, namely to solve cases of apparent homoallelism, i.e., when a single peak may represent two normal alleles of the same size or a second allele expanded beyond the range of detection of the assay (Maciel et al., 2001; Smith et al., 2013).
6. **Allele-specific therapies.** The urgency to develop direct therapies to prevent or slow progression of neurodegeneration led some authors to propose the sub-expression of the causative gene as a potential approach (Evers, 2015). In the case of *ataxin-3*, however, depletion of the gene results in cell death, by accumulation of ubiquitinated material, cytoskeletal disorganization and loss of cell adhesion (Alves et al., 2008). Therefore, the development of small interfering RNAs, based on the presence of a SNP that discriminates between wild-type and mutant transcripts, could be an efficient strategy for treatment of MJD. There are several SNPs described flanking the *ATXN3*_CAG repeat; however, for allele-specific therapy, patients must be heterozygous for the target SNP. The protocol optimized here for MJD may be used to identify the most informative SNPs for each population; the same pipeline can be followed to perform similar analysis in other repeat-associated diseases.

## Conclusion

Identification of mutational haplotype backgrounds is key to unravel the mechanisms behind repeat-associated diseases. The strategy proposed was based on the analysis of fast evolving STR markers, placed within stable SNP-based lineages. By using the example of MJD, we have shown the procedure to assess allelic phases of both SNPs and STRs segregating with expanded alleles. Haplotype definition is crucial in dominant diseases, since normal alleles are not on the mutational background where *de novo* expansion(s) took place but are inherited from the non-affected parent. This is also relevant for recessive diseases, since two mutations (expanded alleles) in homozygosity may not share a common ancestral origin. When the gene of interest is on the X chromosome, that allows to infer haplotypes directly in males (e.g., when analyzing the CAG expansion in the *androgen receptor*, responsible for SBMA) (Santos et al., 2014).

## Author Contributions

SM conceived and designed the study. IC, BA, and SM developed the methodology. IC and BA analyzed the genotype. IC and SM drafted the manuscript. JS, AA, and SM critically revised the manuscript. AA and SM obtained funding and supervised the study.

## Conflict of Interest Statement

The authors declare that the research was conducted in the absence of any commercial or financial relationships that could be construed as a potential conflict of interest.

## Footnotes

1 www.ensembl.org

2 https://tandem.bu.edu/trf/trf.html
